# Analysis of the protein related receptor GPR92 in G-cells

**DOI:** 10.3389/fphys.2015.00261

**Published:** 2015-09-23

**Authors:** Amelie T. Rettenberger, Waltraud Schulze, Heinz Breer, Désireé Haid

**Affiliations:** ^1^Institute of Physiology, University of HohenheimStuttgart, Germany; ^2^Institute of Physiology and Biotechnology of Plants, University of HohenheimStuttgart, Germany

**Keywords:** G-cells, LC-MS/MS, receptors, GPR92, protein diet

## Abstract

A continuous assessment of ingested food in the gastric lumen is essential for fine-tuning the digestive activities, including the secretion of the regulatory hormones such as gastrin. It has been proposed that G-cells may be able to sense the amount of ingested proteins and adjust the secretion of gastrin accordingly. Our previous studies have shown that G-cells express suitable receptor types, most notably the peptone-receptor GPR92 and the amino acid receptors GPRC6A and CaSR; however, their relative importance remained unclear. To determine the relative quantity of each receptor type, individual G-cells isolated from the transgenic mouse line mGas-EGFP were analyzed by means of a Liquid Chromatography Tandem-Mass Spectrometry (LC-MS/MS) procedure. The results indicate that the relative amount of receptor protein for GPR92 was much higher than for the receptor types GPRC6A and CaSR. These findings support the notion that the peptone-receptor GPR92 may be particularly relevant for sensing partially digested protein products. This view was supported by the finding that a high-protein diet affected the expression level of the peptone-receptor *GPR92* in the gastric antrum as well as in the circumvallate papillae.

## Introduction

Adequate digestive activities in the stomach, including the secretion of gastric juice is mediated mainly by the peptide hormone gastrin which is released from G-cells located in the gastric antrum and components of the luminal content in particular protein breakdown products elicit the secretion of gastrin (Taylor et al., 1982; Rérat et al., 1985; DelValle and Yamada, 1990). Thereby the release of gastrin is dependent upon the quantity and quality of ingested protein (Rérat et al., 1985). Gastrin in turn stimulates the pepsinogen secretion from chief cells by activating the appropriate receptors (Cooke et al., 1967; Blandizzi et al., 1999) and it also induces the secretion of hydrochloric acid by activation of parietal cells either directly or indirectly via histamine released from enterochromaffin-like cells (Sandvik et al., 1987). In order to match the gastric activities with the amount of dietary protein in the stomach, the release of gastrin has to be fine-tuned according to the amount of luminal protein; this view implies a continuous assessment of ingested food in the gastric lumen by the G-cells. Our previous studies have shown that G-cells may indeed be able to sense protein breakdown products by three protein related receptors; the peptone-receptor GPR92 (also GPR93, LPAR5, IUPHAR: LPA_5_ receptor) responsive to protein breakdown products as well as the receptor types GPRC6A and CaSR responsive to amino acids and calcium, respectively (Haid et al., 2012). Although the three receptors are coexpressed in G-cells, the relative importance of each receptor type for sensing protein breakdown products is totally unclear. Estimating the relative quantity of each receptor type in G-cells may provide some first hints; advanced analytical technologies, such as Tandem-Mass Spectrometry, allow such quantitative analyses. As an alternative approach to assess a possible role of GPR92 in sensing protein breakdown products, the expression of the peptone-receptor GPR92 was monitored upon feeding with a high-protein (HP) diet, based on the recent observations that high-lipid diet affects the expression levels of lipid-related receptors (Chen et al., 2010; Tran et al., 2011).

## Materials and methods

### Mice

Analyses were performed with the wild type mouse strain C57/BL6J purchased from Charles River (Sulzfeld, Germany). In addition the previously described BAC-transgenic mouse line mGas-EGFP was used, which expresses GFP under the control of the gastrin promoter. GFP-positive cells in mGas-EGFP mice were detectable at the base of the gastric antrum. In contrast only few GFP-positive cells were found in the transitional zone and no positive cells in the gastric corpus. The identity of GFP-positive cells as G-cells was proven by fluorescence immunostainings with a specific gastrin antibody in the original paper (Takaishi et al., 2011). Mice were housed with a 12 h light/dark cycle in groups or individually at the Central Unit for Animal Research at the University of Hohenheim and had access to food and water *ad libitum*. For tissue preparations animals were killed by cervical dislocation and subsequent decapitation. Experiments were carried out in accordance with the Council Directive 2010/63EU of the European Parliament and the Council of 22 September 2010 on the protection of animals used for scientific purposes. The work was approved by the Committee on the Ethics of Animal Experiments at the Regierungspräsidium Stuttgart (V318/14 Phy) and the University of Hohenheim Animal Welfare Officer (T125/14 Phy, T126/14 Phy).

### Nutritional experiments

Nutritional approaches were performed with wild type mouse strains C57/BL6J. 8–14 weeks old animals were divided in eight groups, each consisting of 4–5 littermates. Mice were nourished on control diet or fed a high-protein (HP) diet for 2, 21, 35, or 84 days, respectively. As control diet they were fed a standard laboratory chow containing 33% calories from protein (3.06 kcal/g, 58% from carbohydrates and 9% from fat; V1534-300 R/M-H, ssniff Spezialitäten GmbH, Soest, Germany); HP diet contained 59% calories from protein (4.85 kcal/g, 24% from carbohydrates, 17% from fat, V15209-34 EF R/M High-Protein, ssniff Spezialitäten GmbH, Soest, Germany). All mice had free access to food and water and no significant differences in bodyweight were observed in HP-diet fed mice compared to control mice.

### Tissue preparation

For qPCR analyses tongues and stomachs were dissected from freshly decapitated mice and washed in 1X PBS (0.85% NaCl; 1.4 mM KH_2_PO_4_; 8 mM Na_2_HPO_4_, pH 7.4) to remove stomach contents and other remnants. Afterwards the whole stomach was dissected along the border between corpus and antrum tissue, which is clearly distinguishable from other stomach compartments by means of its characteristic appearance and color; the antral part of the stomach was immediately frozen in liquid nitrogen and stored at −70°C until RNA isolation. After this, circumvallate papillae was stumped out of the detached lingual epithelium using hematocrit capillaries (75 μl/D.A. 1.55 mm; Brand, Wertheim, Germany), transferred into collection tubes and immediately frozen in liquid nitrogen. For visualization of intrinsic EGFP-Fluorescence of transgenic mGas-EGFP mice, the stomachs was removed, rinsed in 1X PBS and immersed in 4% ice-cold paraformaldehyde (in 150 mM phosphate buffer, pH 7.4) for 2 h. After fixation the tissue was cryoprotected by incubation in 25% sucrose overnight at 4°C. Finally, the tissue was embedded in Tissue Freezing Medium (Leica Microsystems, Bensheim, Germany) and quickly frozen on liquid nitrogen. Cryosections (5 μm) were generated using a CM3050S cryostat (Leica Microsystems) and adhered to Superfrost Plus microscope slides (Menzel Gläser, Braunschweig, Germany).

### RNA isolation and cDNA synthesis

Total RNA was isolated from the dissected antrum and circumvallate papillae with a NucleoSpin RNA kit (Macherey-Nagel, Düren, Germany) according to the manufacturer's protocol. To insure the complete removal of DNA, a DNase digestion (DNase I, Life Technologies, Carlsbad, CA, USA) step was included. 1500 ng/μl total RNA was reverse transcribed using oligo(dT) primers and SuperScript III Reverse Transcriptase (RT) (Invitrogen, Carlsbad, CA, USA). RNA integrity of each sample was controlled by the amplification of the housekeeping gene for the ribosomal protein L8 (RpL8) with intron-spanning primers to verify the DNA removal.

### Quantitative real-time PCR and statistics

For determination of quantitative changes in mRNA levels qPCR experiments were performed using normalized cDNA from tissues as described above. PCR amplifications were performed on a LightCycler® 480 instrument (96-well version, Roche Diagnostics, Mannheim, Germany). The qPCR reaction mixture (10 μl) consisted of 5 μl KAPA SYBR® FAST (Kapa Biosystems, Wilmington, Massachusetts, USA) and primer sets. The following qPCR protocol was used: 95°C for 2 min, 95°C for 15 s, 60°C for 15 s, 72°C for 10 s with 40 cycles, a melting step by slow heating from 65 to 95°C with +0.5°C per cycle and a final cooling down to 40°C. Each assay included (in triplicate): for receptor genes 4.95 μl of each tested cDNA, for RpL8 a 1:10 cDNA dilution, a non-template control reaction, and a reaction control with KAPA SYBR® FAST and water. For the amplification primers were generated using the following gene sequences: RpL8: NM_012053; GPR92: NM_001163268.1; GPRC6A: NM_153071; CaSR: NM_013803. The used primers were: RpL8 primers: mRpL8 forward, 5′-GTG CCT ACC ACA AGT ACA AGG C-3′; mRpL8 reverse, 5′-CAG TTT TGG TTC CAC GCA GCC G-3′ (224 bp); GPR92 primers: mGPR92 forward, 5′-TCA GTG CCG AGG GTT TCC GTA A-3′, mGPR92 reverse, 5′-GCC GAA TCC TGG GAG CAG TTG-3′ (205 bp); GPRC6A primers: mGPRC6A forward, 5′-TTC TTA CCT GCA CGG GCA TT-3′, mGPRC6A reverse, 5′-AGG TCA GGA ACT TGG CTT CG-3′ (240 bp); CaSR primers: mCaSR forward, 5′-AGC ACT GCG GCT CAT GCT TTC-3′, mCaSR reverse, 5′-TCA GGG CCA GTG GTT GCT G-3′ (193 bp). Melt-curve analysis and agarose gel electrophoresis of the respective qPCR reactions confirmed the presence of a single PCR product in each reaction and changes in the expression of the housekeeping gene over time and between HP and control groups were very low. qPCR analysis were performed in triplicates for each tested gene and the relative amount of transcripts for GPR92, GPRC6A and CaSR was determined by normalizing the crossing points (*C*_*t*_-values) of target genes to the *C*_*t*_-values of the housekeeping gene RpL8 (reference). *C*_*t*_-values were generated by the LightCycler Software 3.5 (Roche Diagnostics). We used two different efficiency corrected calculation methods to analyze on the one hand (1) the relative abundance of mRNA of the different receptor types in normal fed mice and on the other hand (2) the relative changes in mRNA expression levels of GPR92 induced by the HP compared to control mice. For (1) the mean *C*_*t*_-values of each triplicate were normalized to the housekeeping gene RpL8 using the following formula (Livak and Schmittgen, 2001): Δ*Ct_t_* = 2^*Ct*(target)−*Ct*(reference)^. For (2) we used the ΔΔ*C*_*t*_ method (Pfaffl, 2001): ΔΔ*C*_*t*_ = (E(target)^Δ*Ct*(target(control−HP))^)/(E(reference)^Δ*Ct*(reference(control−HP))^). Real time-efficiencies were calculated by the given slopes of standard curves of dilution series of plasmids encoding the three different receptors and the given slopes in the LightCycler Software 3.5 (Roche Diagnostics). Therefore, the relative changes in mRNA expression were calculated as *n*-fold difference of normalized Δ*C*_*t*_-values of HP compared to control mice.

### Visualization of intrinsic EGFP-fluorescence

Cryosections were air-dried, rinsed three times in 1X PBS for 5 min. The sections were counterstained with 4′,6-diamidino-2-phenylindole (DAPI; 1 μg/ml, Sigma Aldrich, Schnelldorf, Germany) for 3 min at room temperature, rinsed with bidest water and finally mounted in MOWIOL (10 % polyvinylalcohol 4-88 (Sigma), 20 % glycerol in 1X PBS).

### Microscopy and photography

Section staining was documented using a Zeiss Axiophot microscope (Carl Zeiss MicroImaging, Jena, Germany). Images were captured using a Zeiss Axiocam for transmitted light and a “Sensi-Cam” CCD camera (PCO imaging, Kelheim, Germany) for fluorescent images. Images were adjusted for contrast in AxioVision LE Rel. 4.3 (Carl Zeiss MicroImaging, Jena, Germany).

### Single cell isolation

For single cell isolation the antrum from freshly decapitated mGas-EGFP mice was dissected from the stomach and immediately transferred to ice-cold medium A (0.39 g NaH_2_PO_4_; 0.89 g Na_2_HPO_4_; 8.401 g NaHCO_3_; 20.45 g NaCl; 1.864 g KCl; 9.91 g Glucose; 59.58 g HEPES; 4.162 g Na-EDTA). Afterwards antrum tissue was transferred to culture dishes with about 200 μl medium A and mechanically sectioned by scalpel and scissor. A solution of 0.025 g Protease from *Streptomyces griseus* (Sigma Aldrich, Schnelldorf, Germany) and 10 ml Medium A was prepared for further procedures and 5 ml of this mixture was transferred into 50 ml centrifugation tubes (Sarstedt, Nümbrecht, Germany) together with the dissected antral tissue fragments and incubated for 30 min at 37°C, while vortexing every 10 min (enzyme digestion I). After a centrifugation step (5 min, 300 g, 18°C) supernatants were removed and the pellet was resuspended in 5 ml of the protease solution and incubated for 10 more minutes at 37°C (enzyme digestion II). Another centrifugation step (5 min, 300 g, 18°C) followed. Supernatants were removed and the pellet was resuspended in 5 ml Hank's Balanced Salt Solution (HBSS, Gibco§, Invitrogen, Karlsruhe Germany). The cell suspension was subsequently filtered through a 40 μm cell strainer (BD Falcon, Dickinson and Company) and again centrifuged for 5 min at 300 g. Finally, the supernatants were removed, the pellets was resuspended in 600 μl preheated Dulbecco's Modified Eagle Medium (37°C, DMEM, Gibco§, Invitrogen, Karlsruhe, Germany) and stored at 37°C until cell picking at the same day. The procedure used here was based on the cell isolation protocol of Nakamura et al. (2010).

### Cell picking

For cell picking the enriched cells (see above) were resuspended in a small amount of DMEM medium to reach a dilution, which allows to pick single cells comfortably. We used hematocrit capillaries (75 μl/D.A. 1.55 mm; Brand, Wertheim, Germany) which were pulled to an exactly matching tip size for G-cells. For this we made use of DMZ-Universal Electrode Puller (Zeitz, Martinsried, Germany) which produces patch-clamp electrodes regulated by a microprocessor. Capillaries were pulled with the following program: P(A): H = 260, F(TH) = 020, S(TH) = 022, t(H) = 012, S(H) = 060, t(F1) = /, F1 = 0, s(F2) = 0, F2 = 0, AD = 010; P(B): H = 180, F(TH) = 105, S(TH) = 075, t(H) = 006, S(H) = 000, t(F1) = 050, F1 = 020, s(F2) = 100, F2 = 030, AD = 055. Capillaries were filled with 4 μl 1X PBS and inserted into a micromanipulator apparatus (Narishige, Tokyo, Japan) coupled to a syringe by a flexible pipe. The fluorescence microscope (Olympus IX70) was coupled with the micromanipulator apparatus to navigate the inserted capillary to the G-cells and subsequently soak in the cells manually by means of the syringe. After picking 80–200 cells the top of the capillary was broken and cells were pushed into a clean PCR-tube and immediately frozen at −70°C.

### Protein preparation for mass spectrometry

One Hundred picked G-cells were dried in a vacuum concentrator (Christ, Germany) and directly lysed in 6 M urea, 2 M thiourea, 10 mM Tris/HCl pH 8. Extracted protein was predigested for 3 h with endoproteinase Lys-C (0.5 μg/μl; Wako Chemicals, Neuss) at room temperature. After 4-fold dilution with 10 mM Tris-HCl (pH 8), samples were digested with 4 μL Sequencing Grade Modified trypsin (0.5 μg μL^−1^; Promega) overnight at 37°C. After overnight digestion, trifluoroacetic acid (TFA) was added (until pH ≤ 3) to stop digestion. Digested peptides were desalted over C18 tip (Rappsilber et al., 2003) and dissolved in 5% acetonitrile, 0.1% trifluoroacetic acid before mass spectrometric analysis.

### LC-MS/MS analysis of tryptic peptides

Tryptic peptide mixtures were analyzed by LC-MS/MS using nanoflow Easy-nLC1000 (Thermo Scientific) as an HPLC-system and an Quadrupole-Orbitrap hybrid mass spectrometer (Q-Exactive Plus, Thermo Scientific) as mass analyzer. Peptides were eluted from a 75 μm × 50 cm analytical column (Thermo Scientific) on a linear gradient running from 4 to 64% acetonitrile in 240 min and sprayed directly into the mass spectrometer. Proteins were identified by MS/MS using information-dependent acquisition of fragmentation spectra of multiple charged peptides. Up to 12 data-dependent MS/MS spectra were acquired for each full-scan spectrum acquired at 70,000 full-width half-maximum resolution. Overall cycle time was ~1 s. Masses of selected tryptic peptides of G-protein coupled receptors GPC6A, CASR, and LPAR5 were used as inclusion list for preferred fragmentation.

### Mass spectrometric data analysis and statistics

Protein identification and ion intensity quantitation was carried out by MaxQuant version 1.4.0.1 (Cox and Mann, 2008). Spectra were matched against the Mouse database (Swissprot Mouse 2013, 51,248 entries) using Andromeda (Cox et al., 2011). Thereby, carbamidomethylation of cysteine was set as a fixed modification; oxidation of methionine as well as acetylation of protein N-termini was set as variable modifications. Mass tolerance for the database search was set to 20 ppm on full scans and 0.5 Da for fragment ions. Multiplicity was set to 1. For label-free quantitation, retention time matching between runs was chosen within a time window of 2 min. Peptide false discovery rate (FDR), protein FDR, and modification site FDR was set to 0.01. Hits to contaminants (e.g., keratins) and reverse hits identified by MaxQuant were excluded from further analysis. All identified peptides including their m/z, charge and scores are listed in Supplementary Table 1. Reported ion intensity values were converted to iBAQ scores for each protein (Schwannhäusser et al., 2011) using MaxQuant. Distribution of receptor proteins and their relative abundance within the tested samples is visualized in Supplementary Figure 1. For comparison of receptor proteins iBAQ scores from the three separate LC-MS/MS analysis of 100 G-cells from one mGas-EGFP mouse were used as abundance rank within each sample. Significant differences were analyzed by the unpaired *t*-test with GraphPad Prism (Graphpad Software, www.graphpad.com). Statistical significance was set at ^*^P < 0.05; ^**^P < 0.005; ^***^P < 0.0001.

## Results

Our previous studies have shown that the amino acid and calcium receptors (GPRC6A, CaSR) and the peptone-receptor (GPR92) are expressed in gastrin cells (Haid et al., 2011, 2012). However, the functional role and the relative importance of the receptor types for sensing proteins in the gastric chyme remain elusive. Assuming that in the stomach a protein-rich food is broken down into protein fragments rather than free amino acids it seems conceivable that the peptone-receptor GPR92 may play a more prominent role.

**Figure 1:**
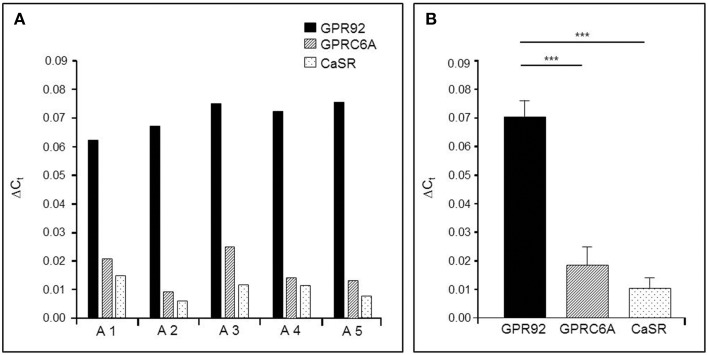
**Quantitative PCR (qPCR) analysis of mRNA for the receptor types GPR92, GPRC6A, and CaSR in the murine gastric antrum from normal fed mice**. **(A)** The relative expression levels of the three receptor types in the antral mucosa from five different mice. In all cases the results indicate the highest expression rates for the peptone-receptor GPR92.**(B)** Mean transcript levels of GPR92, GPRC6A and CaSR from A1-A5 shown in **(A)**. The values for GPR92 are 4.6-fold higher than those for GPRC6A (*P* = 0.004) and 8.1-fold higher than for CaSR (*P* = 0.0003). Ribosomal protein L8 (RpL8) was used as an internal control. Relative expression was calculated using the formula: Δ*C*_*t*_ = E^*Ct*(target)−*Ct*(reference)^ with corresponding efficiencies (E_*GPR*92_: 1.988, E_*GPRC*6*A*_: 1.967 and E_*CaSR*_: 1.965). Data are generated in triplicate are expressed as mean ± SD with *n* = 5 mice. Statistically significant results determined by the unpaired *t*-test (^***^P = 0.001).

In order to get some first insight, we set out to determine the relative expression levels of the three receptor types in tissue samples from the gastric antrum region where G-cells account for 60% of the enteroendocrine cell types (Sachs et al., 1997). The results of quantitative real-time polymerase chain reaction (qPCR) of antral tissues are depicted in Figure 1. The data shown in Figure 1A represent the relative amounts of receptor transcripts in the antrum mucosa determined for five mice. In all cases the highest levels of mRNA were found for the peptone-receptor GPR92; the transcript levels for GPRC6A and CaSR were much lower. This general trend was reflected in the mean transcript levels of the five mice (Figure 1B) showing that mRNA amounts for GPR92 were 4.6-fold higher than for GPRC6A (*P* = 0.0004, Figure 1B) and 8.1-fold higher than for CaSR (*P* = 0.0003; Figure 1B).

**Figure 2:**
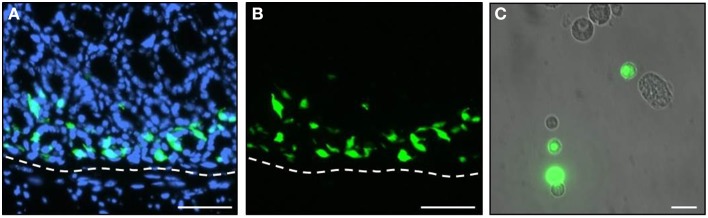
**EGFP-positive G-cells of transgenic mGas-EGFP mice**. **(A,B)** A longitudinal tissue section through the gastric antrum of an mGas-EGFP mouse. G-cells are visible by their strong intrinsic EGFP-fluorescence (green). Sections are counterstained with DAPI (blue). **(C)** After tissue dissociation EGFP-positive G-cells (green) retain their intrinsic fluorescence and can easily be distinguished from non-labeled cell types. Scale bars: **(A,B)**, 50 μm; **(C)**, 20 μm.

Since the mRNA-levels provide only a first approximation concerning the amounts of the actual proteins, attempts were made to determine the relative protein concentration for the three receptor types in G-cells. Such an approach is only possible by means of advanced mass spectrometry methodology and requires isolated cells. Therefore, a procedure was established that allowed to selectively isolating antral G-cells from GFP-BAC transgenic mice (mGas-EGFP) in which G-cells can be identified by their cell-specific strong EGFP-fluorescence (Takaishi et al., 2011). A typical section through the gastric antrum of such a transgenic mouse is depicted in Figures 2A,B; G-cells exhibit a bright intrinsic EGFP-fluorescence. Using these transgenic animals, we have developed a protocol for a mild tissue dissociation which allowed to manually isolate EGFP-positive G-cells by means of micropipettes and micromanipulators under microscopic surveillance (described in the Materials and Methods Section). A typical cell suspension from antral tissue is shown in Figure 2C. Since the EGFP-positive G-cells retain their intrinsic fluorescence during the dissociation process they could be easily distinguished from non-labeled cells and selectively picked out of the cell suspension.

**Figure 3:**
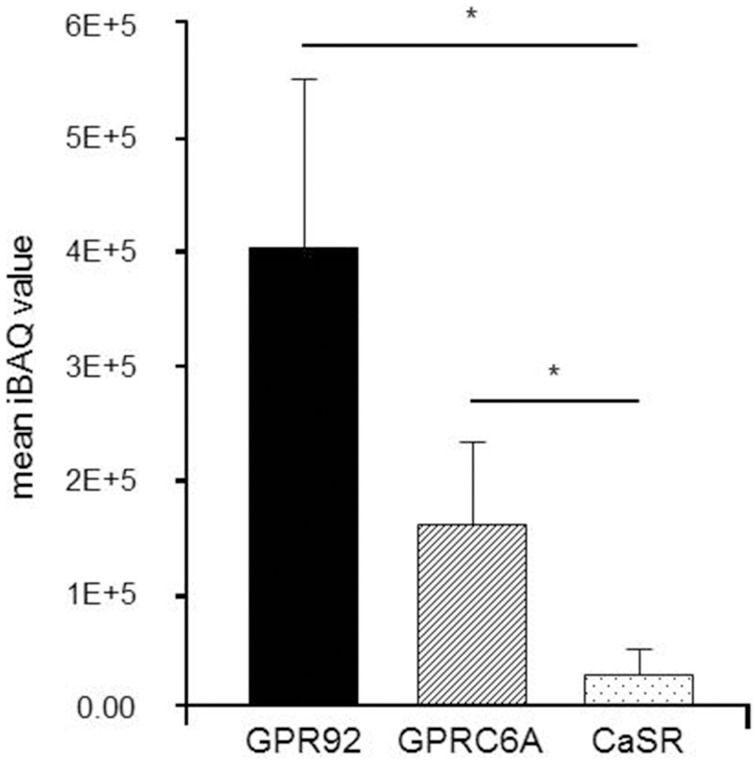
**Label-free relative quantitation of receptor proteins in isolated G-cells from normal fed mGas-EGFP mice**. Liquid Chromatography Tandem-Mass Spectrometry (LC-MS/MS) analyses were performed to determine the relative abundance of receptor proteins in isolated G-cells from one mouse investigated as triplicate. Each run contained 100 G-cells. The results indicate a high abundance of GPR92 receptor protein, whereas GPRC6A and especially CaSR receptor protein levels were rather low. Data are expressed as mean iBAQ scores ± SD (^*^P ≤ 0.05).

The relative amount of proteins in isolated G-cells was determined in a label-free relative quantitation by means of Liquid Chromatography Tandem-Mass Spectrometry (LC-MS/MS). Three replicate runs of protein extracted from 100 isolated cells have led to the identification of 416 proteins. Three receptors GPR92 (three peptides), GPCR6A (six peptides) and CaSR (three peptides) were unambiguously identified with at least three tryptic peptides. For CaSR one, and for GPR92 and GPRC6A two of these peptides were identified in each of the replicates, supported by other peptides that were identified in one or two replicates only (Supplementary Table 2). Sequence coverage was 2.7% for CaSR, 4% for GPRC6A, and 3.6% for GPR92. The quantitative results of this approach are depicted in Figure 3. Databased on the iBAQ abundance values, GPR92 was found as the receptor with the highest abundance while only moderate levels were observed for GPRC6A and CaSR. This finding suggests a prominent role of GPR92 in G-cells.

**Figure 4:**
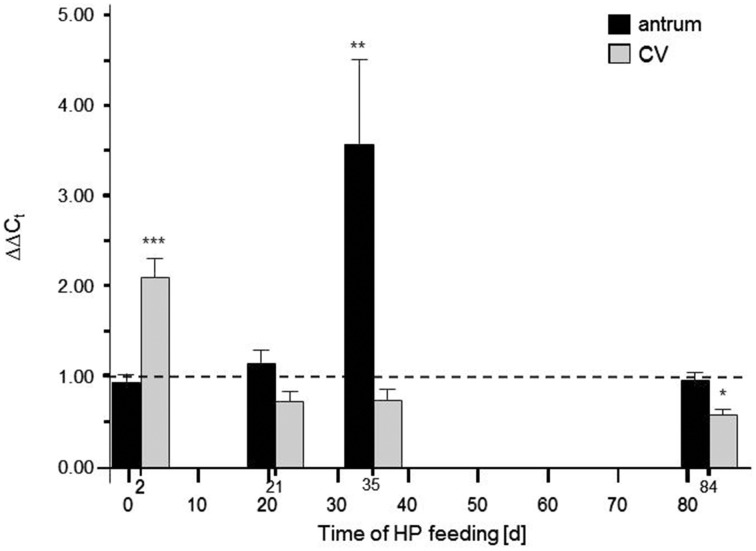
**Effects of high-protein (HP) feeding for different time intervals on the relative amount of mRNA for GPR92 in the murine gastric antrum and circumvallate papillea (CV)**. The results of the qPCR analyses revealed that HP feeding resulted in time-dependent alterations of transcript levels: After 2 days the relative amount of mRNA for *GPR92* were significantly reduced (*P* < 0.001) in the antrum region whereas mRNA quantities in the CV were significantly increased (*P* < 0.001). 21 days of HP feeding did not lead to significant changes of transcript levels. However, after 35 days only in the gastric antrum a strong increase of *GPR92* mRNA amount was observed (*P* = 0.0094). After 84 days the HP diet induced increase of *GPR92* transcripts in the antrum was no longer detectable. Instead, there was a decrease of circumvallate *GPR92* mRNA level (*P* = 0.0493). Relative expressions were calculated using the formula: ΔΔ*C*_*t*_ = (E(target)^Δ*Ct*(target(control−*HP*))^)/(E(reference)^Δ*Ct*(reference(control−*HP*))^) with corresponding efficiencies (E_*GPR*92_: 1.988, E_*GPRC*6*A*_: 1.967 and E_*CaSR*_: 1.965). Data are expressed as mean ± S.E.M. Dashed line denotes baseline levels corresponding to no relative changes of mRNA expression (values = 1). Data were generated in triplicate with *n* = 4−5 mice in each group. Significant results were determined by the unpaired *t*-test (^*^P < 0.05; ^**^P < 0.005; ^***^P < 0.001 against baseline level).

As an independent approach to assess a possible role of GPR92 in protein sensing of G-cells it was hypothesized that a high-protein (HP) diet (59% calories from protein) may have an impact on the expression level of the peptone-receptor *GPR92*. Toward this goal, by means of real-time quantitative PCR (qPCR), the amounts of receptor mRNA were determined in antral tissue samples from mice which were fed the HP diet for different time periods. In addition, the transcript levels for *GPR92* in the circumvallate papilla were determined, where the peptone-receptor is expressed in taste sensory cells (Haid et al., 2013). The results are depicted in Figure 4; it is evident that in the presence of high luminal protein concentration the relative amounts of mRNA for *GPR92* changed over time. During a period of 2 days the level of transcripts for *GPR92* in the circumvallate papillae was significantly increased in HP fed mice compared to controls (^***^P < 0.001) while no change was observed in antral tissue. In contrast after 35 days the relative amount of mRNA for *GPR92* in the antrum was strongly increased in HP fed mice (^**^P = 0.0094). No difference was observed in the circumvallate papillae. After a period of 84 days the relative amount of mRNA for *GPR92* was back to baseline level. Together the results demonstrate that the expression of the peptone-receptor *GPR92* in in the stomach and in the taste buds was affected by feeding a protein enriched diet, but seems to be controlled by different parameters.

## Discussion

G-cells produce the peptide hormone gastrin which is secreted according to the amount of protein within the ingested food. As a first step toward an understanding of this protein-related secretion of gastrin we have previously shown that G-cells express receptor types that potentially interact with protein breakdown products; the peptone receptor GPR92, the amino acid receptor GPRC6A and the calcium sensing receptor CaSR (Haid et al., 2011, 2012). However, so far it was not possible to gain information concerning the relative amount of the receptor proteins and thereby about the relative importance of the various receptor types. In the present study attempts were made to determine the relative quantity of receptor proteins in isolated G-cells by means of LC-MS/MS analysis. The results indicate that G-cells contain a markedly higher amount of the peptone-receptor GPR92 than of the receptors GPRC6A and CaSR. These findings are considered as indicative for a prominent role of the peptone-receptor in G-cells. This notion is in line with the capability of GPR92 to be activated by protein fragments (Choi et al., 2007a,b), and protein fragments are supposed to be abundantly present in the luminal content of the stomach due to the pre-digestion of ingested protein. Moreover, the view that the GPR92 receptor may play a major role in monitoring the protein content is supported by the previous findings that protein fragments are more effective in stimulating gastrin secretion than amino acids (McArthur et al., 1983; Saffouri et al., 1984). The functional implication of the amino acid receptor GPRC6A and the calcium sensing receptor CaSR in G-cells remains elusive. It is possible that they monitor the level of amino acids or Ca2+-ions in the luminal content and thus contribute to sense the protein levels in the lumen. However, it is also conceivable that both are relevant for sensing the concentration of their ligands in the extracellular fluid. More detailed analysis concerning the topographic localization of the receptor types on the surface of G-cells (apical vs. basolateral) may provide some further insight.

Receptors for amino acids as well as for peptone are expressed both on the tongue and in the gastrointestinal mucosa (Bystrova et al., 2010; Haid et al., 2012, 2013; Symonds et al., 2015) and are supposed to play an important role in the regulation of ingestion and digestion. In previous studies it has been shown that in the taste system the expression of receptors is regulated upon exposure to different concentrations of nutrients. For example, it was found that in circumvallate papillae the levels of mRNA for the fatty acid receptor GPR120 as well as for CD36 were changed depending on the concentration of lipids in the diet (Martin et al., 2011). Moreover, a high-fat diet caused a significantly reduced level of mRNA for the taste receptor subunit T1R3 in taste buds (Chen et al., 2010). Also for the intestinal system it was found that a high-fat diet induced a change in the expression level of CD36 (Tran et al., 2011). In line with these observations, an interesting finding of the present study concerns the concentration of mRNA for GPR92 in cells of the antrum as well in the taste papillae. High-protein feeding induced significantly higher levels of *GPR92* mRNA already after a short period of time in circumvallate papillae whereas changes in the antrum occurred only on a medium-to-long turn scale. The causes and consequences of these observations are elusive. In this context, it is interesting to note, that the expression of the lipid-sensor candidates CD36 in the mouse circumvallate papillae was subjected to a short-term, lipid-mediated regulation (Martin et al., 2011). The functional implications of the elevated *GPR92* levels for the taste system are unclear but could either be a higher number of GPR92 receptors in the taste cells thereby increasing the sensory reactivity or may reflect a higher turnover of receptor protein due to a more extensive desensitization. In any case, the protein-induced modulation of GPR92 expression may affect the oro-sensory perception of dietary proteins.

The delayed medium-term elevation of *GPR92* mRNA levels in the gastric antrum region is apparently based on other mechanisms and involved in a different functional context. It is most likely that the high-protein diet immediately requires the release of more gastrin from G-cells; this could be mediated by a variety of mechanisms, including neuronal stimuli (Debas and Carvajal, 1994; Ericsson et al., 2010; Schubert et al., 1992). One could imagine that the medium-to-long term change in mRNA expression reflects a sustained adaption of the system; in this context, the higher level of mRNA may infact reflect a high number of cells expressing GPR92 which are primarily G-cells. This would be in line with a recent study demonstrating that high fat diet lead to elevated levels of mRNA for GPR120, the receptor for long chain fatty acid after several weeks which coincided with and probably reflected a significant increase in the number of GPR120 positive cells (Widmayer et al., 2015).

### Conflict of interest statement

The authors declare that the research was conducted in the absence of any commercial or financial relationships that could be construed as a potential conflict of interest.

## Supplementary material

Supplementary Figure 1 **Abundance distribution of receptor peptides**. Left axis: iBAQ-values for all quantified proteins. Colored dots indicate either membrane proteins (red) or the membrane proteins of the GPCR family (cyan). The abundance distribution of membrane proteins is not different from the soluble proteins, i.e., covering a range of at least four magnitudes. Right axis: sequence coverage of the quantified proteins.

Supplementary Table 1 **List of all identified proteins of mass spectrometric analysis of G-cells fractions as revealed by MaxQuant**.

Supplementary Table 2 **Overview of identified peptides from GPCRs in different replicates**.

## Abbreviations

DAPI: 4,6-diamidino-2-phenylindole
GPCR: G protein-coupled receptor
GPR92: G protein-coupled receptor 92 (GPR93; LPAR5)
GPRC6A: G protein-coupled receptor family C subtype 6A
CaSR: Calcium sensing receptor.
