# Supplementary figures and images for: Analysis of the protein related receptor GPR92 in G-cells
Source: Front Physiol. 2015 Sep 23;6:261. doi: 10.3389/fphys.2015.00261 (PMC4585063; doi:10.3389/fphys.2015.00261)

abundance distribution

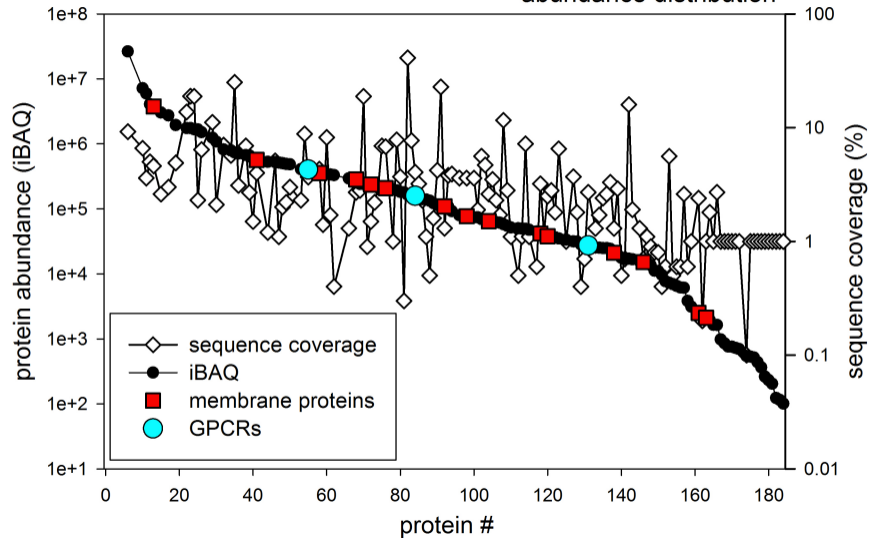

Supplement: Supplementary Figure 1 — Abundance distribution of receptor peptides. Left axis: iBAQ-values for all quantified proteins. Colored dots indicate either membrane proteins (red) or the membrane proteins of the GPCR family (cyan). The abundance distribution of membrane proteins is not different from the soluble proteins, i.e., covering a range of at least four magnitudes. Right axis: sequence coverage of the quantified proteins. [file Image1.PDF]
